# Graphene-Nanodiamond Heterostructures and their application to High Current Devices

**DOI:** 10.1038/srep13771

**Published:** 2015-09-09

**Authors:** Fang Zhao, Andrei Vrajitoarea, Qi Jiang, Xiaoyu Han, Aysha Chaudhary, Joseph O. Welch, Richard B. Jackman

**Affiliations:** 1 London Centre for Nanotechnology, Department of Electronic and Electrical Engineering, University College London, 17-19 Gordon Street, London WC1H 0AH, United Kingdom

## Abstract

Graphene on hydrogen terminated monolayer nanodiamond heterostructures provides a new way to improve carrier transport characteristics of the graphene, offering up to 60% improvement when compared with similar graphene on SiO_2_/Si substrates. These heterostructures offers excellent current-carrying abilities whilst offering the prospect of a fast, low cost and easy methodology for device applications. The use of ND monolayers is also a compatible technology for the support of large area graphene films. The nature of the C-H bonds between graphene and H-terminated NDs strongly influences the electronic character of the heterostructure, creating effective charge redistribution within the system. Field effect transistors (FETs) have been fabricated based on this novel herterostructure to demonstrate device characteristics and the potential of this approach.

Since graphene was first mechanically exfoliated one decade ago^1^, 2D materials have attracted enormous worldwide interest^2^,^3^. In addition to graphene, transition metal dichalcogenides (TMDs)^4^, hexagonal boron nitride (h-BN)^5^ and recently exfoliated phosphorene^3^ enrich the 2D material family. However, none of them exhibit the extreme properties of graphene, such as high thermal conductivity^6^, high mechanical strength^7^, high carrier mobility^8^ and the ability to integrate with most substrates^9^. However, a limitation to the exploitation of graphene as an electronic material is the near-zero bandgap, which results in a small on-off ratio for transistor devices fabricated from this material^10^,^11^. Several approaches have been explored to overcome this problem, such as nanoribbon fabrication, graphene hydrogenation and the use of bilayer graphene; these may create a sizable bandgap but also severely degrade the electronic properties of graphene^12^,^13^,^14^,^15^,^16^. An alternative approach is to modify the supporting substrate material which must inevitably be used when a 2D material is used to fabricated practical devices: The aim being to modify the graphene in terms of band gap creation and doping without severely degrading the carrier transport properties of the graphene layer itself^2^,^17^. Previous studies have typically employed readily available Si/SiO_2_ wafers as the substrate, which creates a thermal capacity problem reducing the current capacity of graphene due to the highly thermal resistive SiO_2_ layer^18^. In an attempt to overcome this, diamond and diamond-like-carbon (DLC) support materials have been explored as they offer electrically insulating properties whilst being superior in terms of thermal conductivity, with a large optical phonon energy and potentially a lower surface trap density than SiO_2_^17^,^19^. Graphene devices on ultrananocrystalline diamond (UNCD) and single crystal diamond (SCD) have been shown to increase the current that graphene on SiO_2_ devices are capable of handling^19^. Nanodiamond particles (NDs), fabricated by a detonation process (being ~5 nm in size) are readily available at low cost and can be easily attached to any 2D or 3D materials through simple sonication from solution^20^; this offers an advantage when compared to the plasma-enhanced chemical vapour depostion (CVD) growth processes required for UNCD and SCD fabrication. Further, ND layers can be readily attached to 2D or 3D substrates over large areas, when compared to plasma-CVD grown materials. NDs also inherit most of the outstanding properties of bulk diamond, whilst delivering them at the nanoscale, including hardness, chemical stability, electrical resistivity and a large bandgap^20^,^21^,^22^. Although not as high as SCD, the thermal conductivity of ND, which is between 5–50 W/mK^23^, is significantly higher than SiO_2_, and also higher than the previously used UNCD (8.6–16.6 W/mK) and DLC (0.2–3.5 W/mK)^24^,^25^. This gives ND layers an advantage over all other graphene supporting materials, in terms of overcoming the thermal capacity issue. In addition, hydrogen termination of ND surfaces can further increase the surface optical phonon energy and stabilize the nanostructures^26^,^27^,^28^.

In this paper, a cost effective and mass producible method to fabricate monolayer ND films that are capable of tuning the properties of graphene for the fabrication of Field-Effect Transistors (FETs) is demonstrated. Compared to similar graphene transferred onto SiO_2_/Si substrates, the mobility increased by 60% when the graphene was deposited on hydrogen terminated NDs (H-NDs). The detailed material properties of graphene on ND surfaces with and without hydrogen termination treatments have been investigated using Fourier transform infrared spectroscopy (FTIR) and Raman spectroscopy. It has been shown that the hydrogen termination treatment not only removed surface contamination from as-deposited monolayer NDs, but also provided a suitable linkage between ND and the graphene layer to form a conductive path, as is demonstrated by impedance spectroscopy (IS) measurements. Also, here the carrier mobility of graphene on hydrogen terminated ND (GrHND) has been compared to graphene on hydrogen terminated SCD (GrHSCD). In addition, to Hall mobility measurements, top-gate graphene transistors with two different gate lengths of 200 nm and 500 nm have been fabricated using a focused ion beam (FIB) tool; and thus, this study has demonstrated a new approach for commercial graphene transistor fabrication.

## Results

### Characterisation of Graphene H-terminated Nanodiamond heterostructures

**Figure 1:**
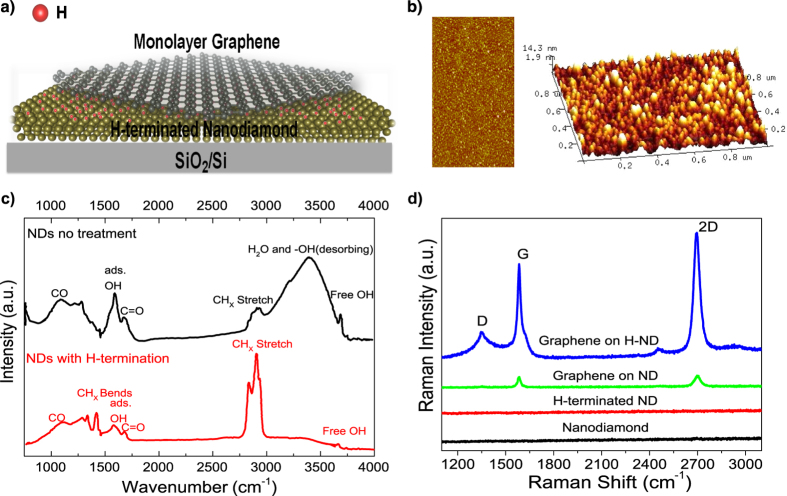
Schematic illustration of the GrHND and structural characterization. (**a**) Schematic view of a graphene-HND heterostructure, Nanodiamond with hydrogen termination. (**b**) AFM images of top-view and 3D-view of hydrogen terminated ND surface. (**c**) Comparison of FTIR spectra of untreated ND and hydrogen-terminated ND surfaces. (**d**) Raman spectra of untreated ND, H-ND, graphene-ND, and graphene-HND.

A schematic view of the GrHND heterostructures supported on a SiO_2_/Si substrate (300 nm SiO_2_) is shown in Fig. 1a. The ND monolayer fabrication included only two simple processes, namely, coating the SiO_2_/Si substrate with an ND-water solution, then an ultra-sonic bath treatment to attach the NDs, followed by drying. Thermal hydrogenation was then performed (detailed process parameters are given in the experimental section). The surface coverage and roughness of the ND layers were determined by atomic force microscopy (AFM), as shown in Fig. 1b in a top-view (1 μm × 0.5 μm) and a 3D-view (1 μm × 1 μm) respectively. A homogeneous layer of ND film has been formed on the substrate by the ND nanoparticles that are 10–20 nm in size. The mean surface roughness is around 2.7 nm and the height of the ND layer suggests it is a monolayer ND film. The uniformity of the deposited ND films is also important as the electronic properties of graphene can be easily modified by the surface morphology of supporting materials such as physical defects, local strain *etc*^29^. The hydrogen termination treatment used here aims to reduce the density of surface states and stabilize the nanostructures^20^,^26^. The Fourier Transform Infrared spectroscopy (FTIR) spectra of untreated ND and H-ND are shown in Fig. 1c. The peak observed at 1726 cm^−1^ is characteristic of the C = O stretching band involved in carboxylic acid groups and anhydride functionalities^30^. The wide peak in the 3000–3600 cm^−1^ region is attributed to the OH of adsorbed water. Various peaks relating to other ND surface groups (C-OH, COO-, C-O-C, C-H) are observed in the 1000–1500 cm^−1^ range^31^. These indicate that the otherwise expected dangling bonds on the surface of the ND films are reactive with air at room temperature. After hydrogen treatment, the intensities of all of the complex mixed peaks from 1000 to 1500 cm^−1^ have been reduced. The free and adsorbed -OH peaks have disappeared in the region of 3000–3600 cm^−1^, while the peak at 2923 cm^−1^ has become much stronger, being assigned to the C-H stretching of the hydrogenated ND surface coupled with the CH_X_ bands at 1461 cm^−1^ (CH_2_) and 1377 cm^−1^ (CH_3_)^32^.

Raman spectroscopy was next used to study the graphene-ND hetrostructure to investigate the chemical nature of the carbon materials present^33^,^34^. The incident power was kept to <1 mW to avoid sample damage and to minimize laser-induced heating^35^. Figure 1d shows the Raman spectra for ND, H-ND films and the two types of graphene-ND heterostructures; the Raman spectra for SCD, H-SCD, GrSCD and GrHSCD are shown in the supplementary Fig. 1. The three main peaks identifying the vibrational modes of graphene are notated as the D, G and 2D peaks, as labelled in Fig. 1d. The G peak assigns to the E_2g_ phonon at the Brillouin zone centre and the D peak is due to the breathing modes of an sp^2^ phase and defects for those activations^36^. The most prominent Raman feature of graphene is the 2D peak whose shape can be used to distinguish single and multilayer graphene^37^. As expected, no Raman peaks can be identified from monolayer dispersed ND and H-ND surfaces due to the low incident power and strong fluorescence generated by the green laser source (514 nm) from these materials^38^. The detailed positions of D, G and 2D peaks are listed for reference in the supplementary Table 1. Deposition of the 2D graphene onto a substrate leads to unavoidable impurity charge doping, and in the case of the use of diamond materials, a mixed sp^3^/sp^2^ bonded phase can be expected for graphene on NDs and H-NDs. The dangling bonds from ND and H-ND surfaces evidenced in the FTIR results lead to the observed shifts in the Raman peaks as compared to unsupported graphene layers^39^,^40^. In particular, the G peak position has been observed to strongly depend on the interaction between graphene and its environment^41^. Compared with previous studies of graphene supported on SiO_2_, here, the G peak positions for the graphene-ND heterostructures have an upshift from 1581 cm^−1^ to 1586.2 cm^−1^ and 1585.7 cm^−1^ for untreated ND and H-ND substrates respectively^34^. These G peak shifts are due to the electron-phonon coupling which results in a shift in the Fermi level, thus doping of graphene^41^,^42^. The 2D peak positions are slightly different on the two different substrates and have downshifts by 2 and 10 cm^−1^ for ND (2698 cm^−1^) and H-ND (2690 cm^−1^) substrates, respectively, due to hole doping^43^, compared with the 2700 cm^−1^ 2D peak of graphene on SiO_2_. The 2D peak widths for both heterostructures are within 25–28 cm^−1^ range, suggesting the graphene is monolayer in nature^35^. Additionally, since the 2D peak responds to all electron scattering processes while the G peak is not affected by such processes, the integrated intensity ratio of I_2D_/I_G_ can be used to provide additional information^37^. The impurity charges and electron transfer induce an increase of scattering with increased carrier concentration, decreasing the ratio of I_2D_/I_G_^34^. The ratios of I_2D_/I_G_ for both heterostructures are 1.06 and 1.17 respectively, proving electron transfer between the interface and thus high levels of doping of the graphene has occurred; for low doping the 2D peak is expected to be 3–5 times stronger than the G peak^34^. The first order D peak can only be observed from Gr-HND, indicating the C-H stretching leads to the appearance of the D peak. The emergence of the D peak is associated with disorder and the sp^3^/sp^2^ ratio^37^, which is in line with previous studies conducted on hydrogenated graphene layers^15^. As observed previously from the FTIR results, the strong C-H stretching for H-terminated sample is responsible for the sp^3^ signal, leading to the heterostructure here being formed from sp^3^/sp^2^ carbon mixtures. Both FTIR and Raman results show a clear modification occurs to the graphene lattice with H-termination treatment of the NDs. The charge transfer between graphene and NDs introduces disorder and high doping to graphene, hence tuning the graphene electronic properties.

**Figure 2:**
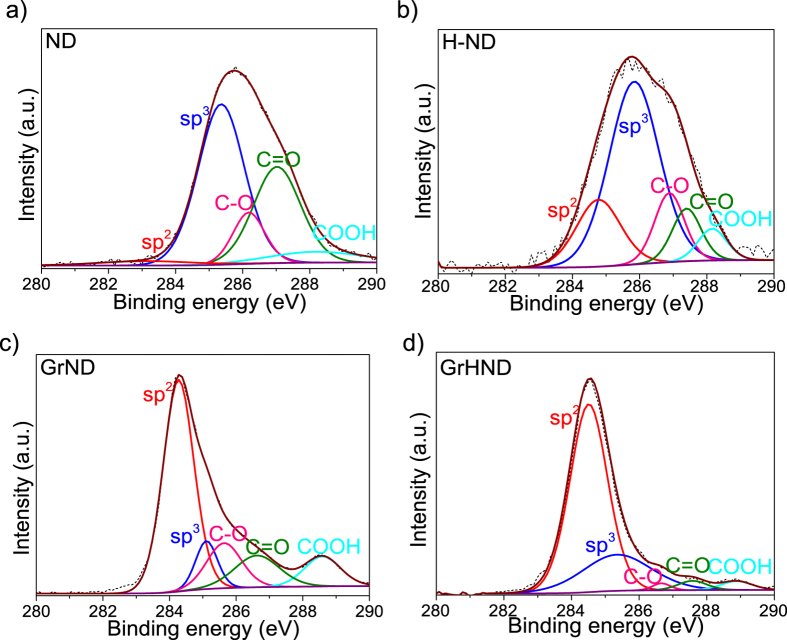
High resolution C1s XPS spectra of different substrates. (**a**) ND. (**b**) H-terminated ND. (**c**) graphene on ND. (**d**) graphene on H-terminated ND. (sp^2^ peak red line, sp^3^ peak blue line, C–O peak pink line, C = O peak green peak, COOH peak cyan line.)

XPS was used to estimate the fraction of sp^2^ and sp^3^ atoms present^44^. Figure 2 shows the C 1s spectral region for ND, H-ND surfaces and for each with graphene overlayers. The C 1 s core-level spectra of samples were fitted by CasaXPS software with the following reference peaks: 284.5 eV binding energy (BE) assigned to sp^2^ hybridized C atoms in ND (graphite structure) and graphene^44^,^45^; 285.4 eV BE assigned to sp^3^ hybridized C atoms due to the formation of C-H and the C–C bonds in the ND film^44^,^46^; other components including C–O, C = O, and COOH with much higher BEs (286.3 eV, 287.6 eV, 288.6 eV) which originated from interface states and ambient oxygen and moisture^46^,^47^,^48^. The XPS spectrum of ND films shown in Fig. 2a, indicates an oxygenated surface with different functional groups due to oxidation in air. At the same time, there is also a small sp^2^ contribution since NDs are partially covered by a thin layer of graphite^20^. Hydrogen termination of NDs (Fig. 2b) reveals the sp^3^ hybridization of the deposited carbon by saturation with hydrogen atoms, whilst thermal annealing (500 C for 5 hours for H-termination as detailed in the experimental section) leads to graphitization and an increase of the sp^2^ phase^46^,^49^. As shown in the previously described FTIR results, most oxygen functional groups were reduced by hydrogen termination; the XPS results confirm that after transferring graphene, oxygen functional groups are more significant on GrND heterostructures (Fig. 2c) rather than GrHND heterostructures (Fig. 2d). When taking into account the D peak assignment in the Raman analysis, these XPS results indicate disorder and the absence of an sp^3^ phase contributing to the D peak^14^,^15^. The C-H stretching sp^3^ bonds are likely to affect the translational symmetry of C = C sp^2^ bonds in graphene, which is similar to the effect of hydrogenating graphene^15^. To analyse the hydrogen coverage first the sp^3^ coverage was determined as θ/(1 + θ) where θ is defined as the intensity area of sp^3^ and sp^2^ peaks^15^. For pristine CVD graphene transferred onto untreated ND films the sp^3^ coverage was found to be ~19%, while for graphene transferred on H-ND films the sp^3^ coverage increased to ~37%. As a result, the hydrogen terminated ND film has also indirectly hydrogenated the monolayer graphene to an H-coverage of approximately 37%−19% = 18%. (Detailed data for sp^2^ and sp^3^ levels is included in the supplementary Table 2).

### Electronic transports in Graphene-Nanodiamond heterostructures

**Figure 3:**
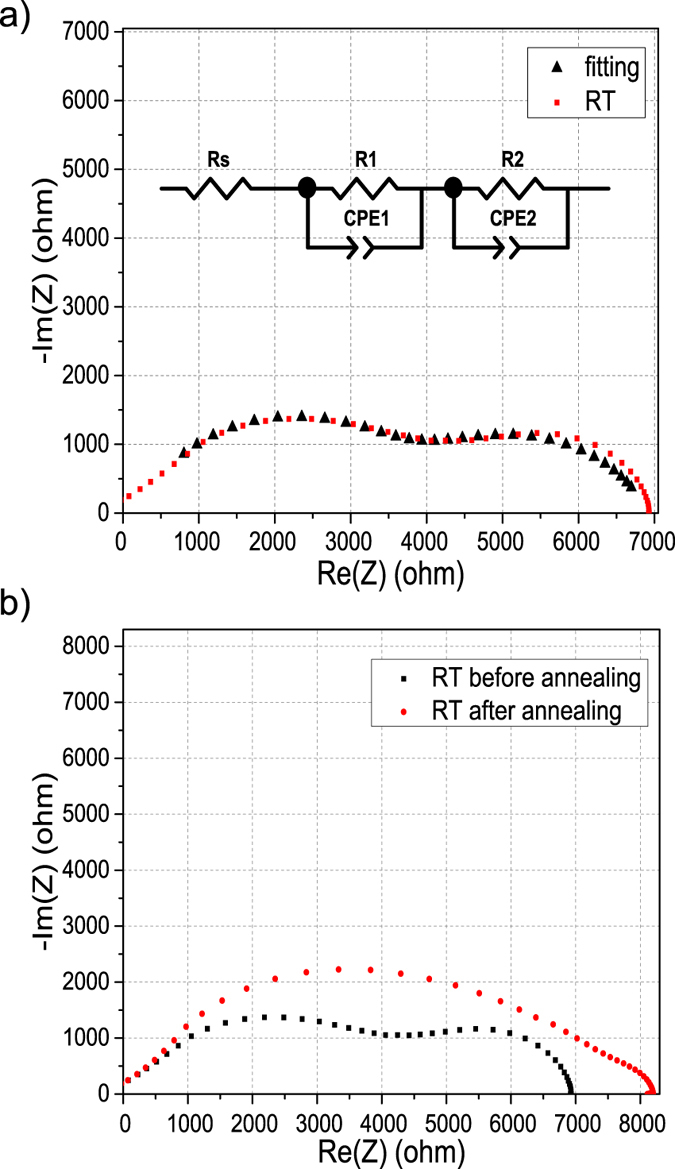
Impedance characterization of GrHND. (**a**) Impedance spectra of GrHND heterostructure (red line). The inset is the equivalent circuit model and the fitting line in black. (**b**) Comparison of room temperature Impedance spectra before and after annealing.

**Figure 4:**
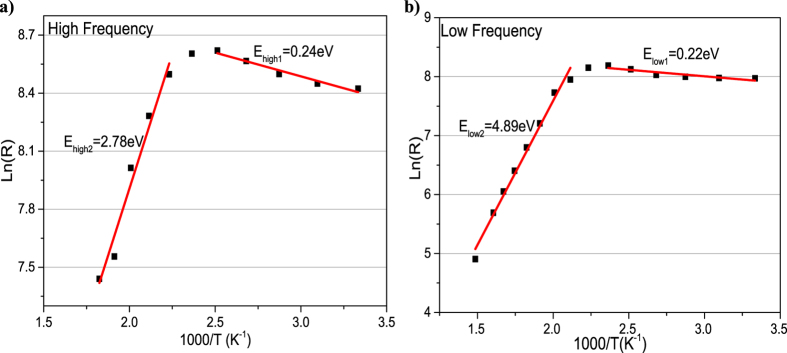
Arrhenius plots of logarithmic sheet resistance and activation energies (a) for high frequency semicircles led by C–H bonds; (b) for low frequency semicircles led by graphene layer.

The electrical characteristics of the heterostructures were examined using AC impedance spectroscopy (IS) measurements. Two Au/Ti (300 nm/10 nm) contacts were deposited on the top-side corners of square samples. IS was carried out in vacuum at temperatures ranging from room temperature to 500 C as shown in Fig. 3 (more details for different temperatures are given in the supplementary Fig. 2). At room temperature the Cole-Cole plot (real component of the impedance plotted against the imaginary part, as a function of frequency) consists of two semicircles, which suggests two conduction paths in the heterostructure^50^. One conduction path is likely to be associated with the path that the charge carriers take at the surface of graphene in contact with the exterior. The other path can be associated with the graphene-nanodiamond interface, where the two carbon surfaces are weakly linked by hydrogen bonds. The sheet resistances for different temperature semicircles are shown in the supplementary Table 3. From the impedance data given in the supplementary information, when temperatures are under 125 C, the radius for the low frequency semicircle increases as the temperature increases, while the high frequency semicircle shows no response to temperature (supplementary Fig. 2a). At higher temperatures, 150 C to 250 C, both semicircles start to decrease (supplementary Fig. 2b). For the low frequency semicircle this might be due to the elimination of residues or remaining PMMA on the graphene surface, which would explain its increasing conductivity. In contrast, for the high frequency semicircle, this can be explained in terms of activation of the hydrogen bonds at the GrHND interface. With a continuing increase in temperature, the high frequency semicircle decreases at a higher rate than the other, and results show that after 275 C there is only one semicircle that further decreases up to 500 C (supplementary Fig. 2c). This suggests the low-frequency semicircle can be assigned to the graphene surface conduction path, while the high-frequency semicircle is explained in terms of a possible activation of the hydrogen bonds in the graphene-ND interface^51^,^52^. The equivalent circuit model of this heterostructure is also shown in Fig. 3a as an inset, representing the internal resistance for the conductive path according to established literature^50^,^53^,^54^. The equivalent circuit model shows two RC in series and the black line in Fig. 3a is the fit resulting from this model. In the equivalent electrical circuit, Rs (209 Ω) represents the total resistance of the electrolyte and electrodes; R1 (3632 Ω) is assigned to the interface linked by H-bonds; R2 (3054 Ω) is from the graphene surface. The conduction path is complicated due to the H-terminated nanodiamond surface. Although the H-terminated ND surface is expected to be conductive, the resistance expected from this kind of mechanism is very high; Su *et al.* found it is around 2.46 × 10^5^ Ω^55^. After high temperature annealing (during measurement) and cooling to room temperature again, two clear semicircles were then not seen, due to the breaking of C-H bonds between the GrHND interfaces at the highest temperatures (Fig. 3b). As shown in the following Hall Effect section, the effect of high temperature annealing of GrHND leads to a mobility value similar to that for GrND (supplementary Table 4). Figure 4 shows the ln(R) vs. 1000/T plot at various temperatures. This suggests a thermally activated conduction process is occurring in the heterostructures. The relevant activation energy is depicted by the Arrhenius equation R = A_0_exp(−E_a_/K_B_T), where A_0_ is the pre-exponential factor; K_B_ is the Boltzmann constant and E_a_ is the activation energy representing the energy barrier for the thermal activation process^55^,^56^. From room temperature to 150 C, the resistances for both semicircles increase with increasing temperature, and the activation energies for low frequency and high frequency semicircles are 0.22 eV and 0.24 eV respectively, which suggests at relatively low temperatures both layers have a similar conduction mechanism, most likely due to a water/adsorbate layer. For high frequency semicircles, induced by the C–H bonds, the resistance decreases following the increase of temperature from 175 C until 275 C at which point the high frequency semicircle disappears; this is considered to be due to disruption of the C–H bonds, displaying activation energy of 2.78 eV. Whilst the resistances of the semicircles attributed directly to the graphene layer keep decreasing from 175 C to 500 C; the activation energy is now 4.89 eV. The large difference of activation energies for high temperatures suggests different carrier transfer mechanisms for the two layers, being caused by thermal expansion and chemical (and/or adsorbate) modification. Therefore, there are two conduction mechanisms for the GrHND heterostructures shown in the equivalent circuit model with two RC in series.

**Figure 5:**
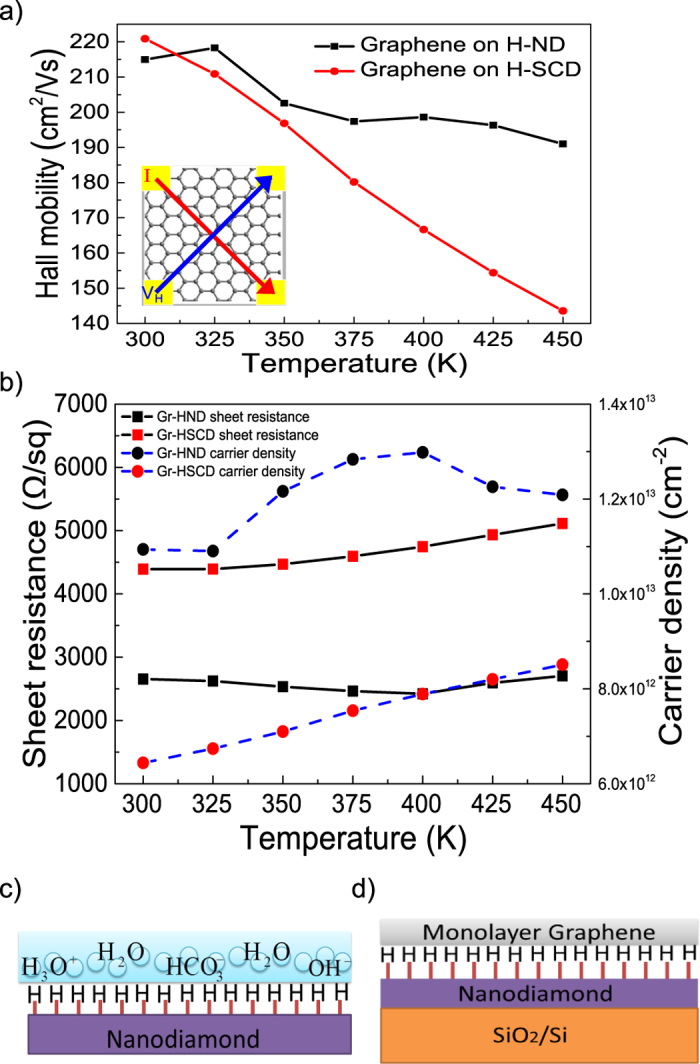
Hall properties of GrHND and GrHSCD. (**a**) Hall mobility for GrHND and GrHSCD. The inset is Van der Pauw method for measurement. (**b**) Comparison of sheet resistance and carrier density of GrHND and GrHSCD. (**c**) Schematic picture of the hydrogenated ND surface in contact with a water layer as it forms in air. (**d**) Schematic for GrHND heterostructure.

Hall effect measurements can be used to determine sheet resistivity, carrier density and Hall mobility values for a semiconductor^57^. The carrier transport properties of GrHND samples were investigated using the Van der Pauw method, as shown in the inset of Fig. 5a. The sample was placed on an insulated platform where four ohmic electrical contacts were deposited on four corners, being Au/Ti (300 nm/10 nm). Here the two different heterostructures are compared: GrHND and GrHSCD. The mean surface roughness of HSCD is 0.61 nm and the AFM images for HSCD are shown in the supplementary Fig. 3. For the reference sample, the mobility for graphene transferred directly onto SiO_2_/Si is 132 cm^2^/Vs (supplementary Table 4). It can be seen from Fig. 5a, the Hall mobilities for both samples are comparable at 300 K and higher than the reference sample, the difference is within 10 cm^2^/Vs. As the temperature increases, the Hall mobility of GrHND exceeds that of GrHSCD. The Hall mobility of GrHSCD is proportional to T^−1.1^ and drops sharply from 220 cm^2^/Vs to 140 cm^2^/Vs, whilst in the case of GrHND the mobility only decreases by 15 cm^2^/Vs. At high temperatures, 450 K, GrHND outperformed GrHSCD in terms of mobility by 33%. This phenomenon can be mainly attributed to phonon scattering with increasing temperature. The acoustic phonons and optical phonons have different impacts on the carrier mobility at different temperatures, which can be defined by ~T^−1.5^ and ~T^−0.5^, respectively^58^. The results presented here show that the acoustic phonon interaction dominates in the GrHSCD case. A more complex scattering process model^59^,^60^ such as Matthiessen’s rule suggests all the impurities, phonon, lattice and defects lead to a shortened carrier scattering time^58^. In addition, when the substrate supports an ND layer, structure transformations, such as graphitization of the NDs, can occur when the temperature increases^61^. The graphitized nanodiamond material can then provide additional carriers for the conductivity. This phenomenon is the key factor in the observation that GrHSCD does not display high current capabilities upon heating, whilst GrHND heterostructure offers high mobility and high carrier density at high temperatures. Therefore, it can be anticipated that devices based on GrHND can be operated within a wide temperature range without losing performance. This is a major advantage of this material system.

The sheet resistance and carrier densities for both heterostructures have been plotted against temperature as shown in Fig. 5b. The sheet resistance of GrHND is stable around 2600 **Ω**/sq from 300 K to 450 K whilst the sheet resistance of GrHSCD shows a small increase from 4392 to 5112 **Ω**/sq over the same temperature range. The carrier density for GrHND is nearly four times that of GrHSCD, presumably due to the complex ND structures present compared to the more homogenious SCD system. An ND film includes three different carbon phases: the diamond phase in form of nanosized grains, trans-polyacetylene (TPA) segments, and amorphous carbon at the grain boundaries^62^. After hydrogen termination, hydrogen prefers to incorporate into the amorphous carbon phase at the grain boundaries, which leads high carrier concentrations for the ND layers - even when compared to hydrogen treated SCD subjected to the same for hydrogen termination process. Hence, more free charge carriers are available on the ND surface than SCD surface, so the carrier density of GrHND is higher than that of GrHSCD. The carrier densities of GrHND increased from 1.1 × 10^13^ cm^−2^ to a highest point of 1.3 × 10^13^ cm^−2^ at 400 K, then dropped slightly to 1.2 × 10^13^ cm^−2^ at 450 K. The carrier density of GrHSCD shows a linear increase with temperature from 6.4 × 10^12^ to 8.5 × 10^12^ cm^−2^ at room temperature to 450 K.

Although GrHSCD has lower a carrier density which enables an increase in the mobility^63^, GrHND gives a lower sheet resistance which offers superiority in terms of the potential for a low resistance channel for a transistor^10^. This suggests the low cost monolayer ND is a good alternative as compared with SCD. The electronic properties of annealed GrHND are similar to those for GrND, as shown in the supplementary Table 4. All of the heterostructures are p-type systems. After hydrogen termination, the diamond surface was observed as a p-type semiconductive layer as show in Fig. 5c; this is a well-established observation^64^. Following wet-chemical oxygenation, C-H dangling bands react with H_2_O and CO_2_ to produce H_3_O^+^, HCO_3_^−^ and H^+^ layers shown in Fig. 5c. Hydrogen termination and surface passivation induce a hole accumulation layer on the surface^65^. After graphene transfer onto such a surface, the electrons from the graphene surface inject into the diamond surface and more holes are left on the graphene layer which leads to a p-type surface of graphene, as shown in Fig. 5d.

### Graphene Field Effect Transistor on H-terminated Nanodiamond

**Figure 6:**
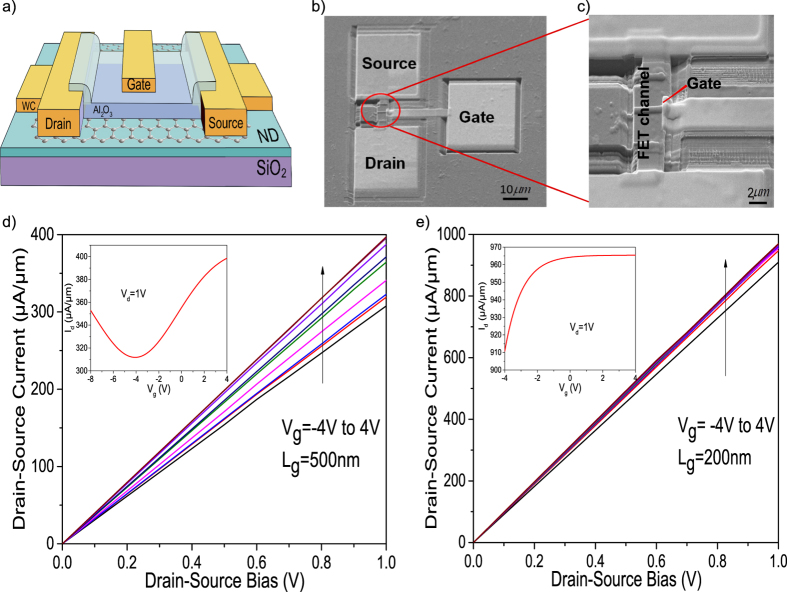
Fabrication and output characteristics for graphene FET on H-terminated ND. (**a**) Schematic view of a top-gate FET. (**b**) SEM image of a top-gate devices, Scale bar, 10 μm. (**c**) FIB image of the 500 nm device. Scale bar, 2 μm. (**d**,**e**) Output characteristics of a 500 nm device (**d**) and a 200 nm device (**e**). Insets, transfer characteristics at drain-source voltage V_ds _= −1 V.

Top gate FET devices were fabricated on GrHND heterostructures with different gate channel sizes of 200 nm and 500 nm, respectively. A focussed-ion-beam tool, giving a potential feature size smaller than 10 nm, was used to define the source, drain contacts, gate electrodes. FIB was also used for local material deposition for contact formation using tungsten carbide (WC). A top-gate Al_2_O_3_ dielectric layer with 10 nm thickness was grown by atomic layer deposition (ALD Ultratech Fuji F200). Detailed fabrication information is given in the fabrication method section. Figure 6a shows a schematic diagram of the fabricated devices and Fig. 6b,c show SEM images of the actual FET design. From Fig. 6c it can be seen that excess graphene was removed by FIB milling around the FET channel to form a sub-micron sized gate. Figure 6d,e show the output characteristics of two graphene devices, one with a gate length of 500 nm and another with a gate length of 200 nm, which show comparable characteristics when compared with the FETs fabricated on GrSCD in Yu’s work^19^. The drain voltage sweeps from 0 to 1 V and the gate voltage changes from −4 V to 4 V. As shown in the figure insets, the Dirac point voltage obtained from the long gate device is around −1 V, which suggests n-doping^48^. However, from the Raman and Hall effect results measured here, the structure is considered p-type. Previously, Li has reported that a graphene FET displayed p-type behaviour due to the Ti layer serving as the hole dopant, as well as the influence of the adsorption of oxygen and water molecules^66^. In the present case, Ti contacts were used in the material studies but WC contacts have been used for the devices because of a lack of availability of Ti/Au contact technology in the FIB system. Both Ti and WC contacts have low resistance and the same difference of work function when compared to the work function of graphene (band diagrams’ comparison for graphene/Ti and graphene/WC contacts in the supplementary Fig. 4). Wu *et al.* measured the Dirac point voltage of their graphene FETs formed on DLC^17^ as about −7 V; they considered this large value to be a result of impurity charge doping during the transfer process. Conversely, Shi *et al.* suggested that the aluminium nanoparticles present in their work tuned the Dirac point, which then induced n-type doping^67^. The inset for the short gate device shows the output characteristics without an inflection point, which suggests that the graphene provides efficient contacts to the conduction band of the NDs for electron injection^68^. The gate modulation of the short gate device is much weaker than that of the long gate device due to the more dominant role of the contact resistance in short gate devices and the short-channel effect^17^. Device mobility was estimated to be μ = (dI_ds_/dV_g_) × (L/WC_i_V_ds_), where L is the channel length (5 μm), W is the channel width (500 nm) and C_i_ is the areal capacitance per unit area between the channel and gate (8 × 10^7^ F/cm^2^)^62^. The mobility for the long gate device is ~149 cm^2^/Vs, which is similar to the measured Hall mobility.

### Concluding remarks

A systematic study of the properties of graphene-ND heterostructures has been carried out and the operation of an FET on GrHND structures demonstrated. Due to the prospects for low cost, easy deposition and an associated high thermal conductivity, monolayer ND is a promising candidate as a support material for graphene. It appears a better solution than other novel materials such as SCD or UNCD in case of the need for high current-carrying device capacity and mass reproducibility. The properties of graphene on NDs with and without H-termination have been investigated using FTIR, Raman spectroscopy, and XPS. The C-H bond present on H-terminated NDs, strongly influences the heterostructure properties that result from depositing graphene on the NDs. The presence of H-termination leads to the appearance of a so-called D peak, and to a shift in the G and 2D peaks, suggesting the ratio of sp^3^/sp^2^ carbon present has changed. From IS measurements performed at various temperatures, it is indicated that the hydrogen links both ND and the graphene layer to create a charge transfer process which induces a conductive interface layer between graphene and ND. The mobility of GrHND increases some 60% compared with similar graphene on SiO_2_/Si and is comparable with observations GrHSCD, which is not viable for commercial applications. Moreover, over the whole temperature range, GrHND demonstrated stable carrier mobility compared to GrHSCD which is vital for commercial applications. Whilst the mobility of the CVD-derived graphene layers here was modest, it can be expected that the advantage of the use of a supported ND layer, over SCD, DLC or SiO_2_/Si would be maintained for higher quality CVD-grown graphene layers as they emerge as commercial materials. FETs fabricated on this novel type of herterostructure GrHND demostrated comparable characteristics to those FETs on graphene-SCD, whilst offering the prospect of low cost large area production of graphene electronics.

## Methods

### Preparation of GrHND heterostructure

As shown in Fig. 1a, the monolayer nanodiamond films were first deposited onto SiO_2_/Si substrates with 300 nm thickness of SiO_2_. Nanodiamonds were supplied prepared in solution from ultra-dispersed detonation nanodiamonds (New metals & Chemicals Corporation, Japan). Deposition involved immersing the substrate material in the ND solution (0.05 g/L of NDs) followed by sonication (10 min). Hydrogen termination for NDs was performed using a Seki AsteX AX6550 reactor with the following parameters for 5 hours: microwave power, 600 W; H_2_ 200 sccm; pressure 10 Torr; temperature 500 C. CVD grown monolayer graphene on a copper foil (Graphene Supermarket) was coated with PMMA. The PMMA-coated graphene was treated with Iron nitrate solution to etch away Cu foil. The floating PMMA coated graphene was removed and cleaned in DI water to transfer onto H-terminated NDs samples. The PMMA was removed by acetone.

### Structural characterization and analysis

The surface morphology of the various films measured over 10 μm × 10 μm and 1 μm × 1 μm regions was measured using scanning atomic force microscopy (AFM, Veeco Dimension V) in tapping mode for the H-terminated NDs. The chemical composition and bonding states of the films were characterized using Fourier transfer infrared spectrometry (FTIR, Perkin Elmer Spectrum One) in transmission mode and X-ray photoelectron spectroscopy (XPS, Thermo Scientific) with the Al K α line as the exciting source. A micro Raman spectroscopy system (Renishaw Invia) operating at 514.5 nm was used to study the film microstructure, where the laser output power used was 1 mW. Impedance spectroscopy was applied in the range of 0.1 Hz to 10 MHz in a vacuum at elevated temperature from room temperature to 500 C using an Autolab electrochemical system. Hall Effect measurements (1T electromagnet, Lakeshore Cryogenics System) were performed to determine graphene carrier mobilities using the “van der Pauw” method, where by four Ohmic metal contacts (10 nm Ti-300 nm Au) were placed on the topside of the graphene layers at room temperature.

### Fabrication of top-gate FET on H-terminated ND

Graphene field-effect transistors were fabricated using a Carl Zeiss Focused Ion Beam Microscope and an Atomic Layer Deposition system. A dual-beam instrument (Carl Zeiss XB1540 cross-beam Focussed-Ion-beam microscope) was used to pattern samples without using masks, giving a feature size as small as 10 nm. The instrument combines a FIB and a SEM column in the same chamber and is fitted with a gas-injection system to allow local material depositions (tungsten carbide, WC, and SiO_2_) and material-specific preferential milling can be performed by introducing reactive gases in the vicinity of the electron or ion probe. The electron column delivers the imaging abilities of the SEM which is less destructive than FIB imaging. SEM imaging of the graphene sample before milling identified clean area suitable for FET fabrication. Keep one clean graphene area 5 μm × 10 μm then other surrounding area around 100 μm × 110 μm were milled by using a 30 kV Ga^+^ beam with a current of 2 nA to remove the graphene and nanodiamond layers for FET definition. Before depositing S/D/G contact patterns, an insulator material (SiO_2_) was deposited on graphene surrounding area (50 nm thickness). Three contacts patterns 40 μm × 40 μm were coated by Tungsten Carbides with thickness of 200 nm using a 30 kV/2 nA beam. A final gentle “polish” with Ga^+^ ions (30 kV/ 5 pA) was used to clean the graphene area edge and remove side damage. Finally connect contact pattern with graphene area edge by WC using a 30 kV/50 pA beam. After the deposition of 10 nm Al_2_O_3_ layer by Atomic layer deposition (ALD), the Al_2_O_3_ layer on big gate pattern was removed then one narrow gate channel was built on top. In this study, the gate sizes are 500 nm × 5 μm and 200 nm × 5 μm.

## Additional Information

**How to cite this article**: Zhao, F. *et al.* Graphene-Nanodiamond Heterostructures and their application to High Current Devices. *Sci. Rep.* **5**, 13771; doi: 10.1038/srep13771 (2015).

## Supplementary Material

Supplementary Information
