# Supplementary material for: Graphene-Nanodiamond Heterostructures and their application to High Current Devices
Source: Sci Rep. 2015 Sep 9;5:13771. doi: 10.1038/srep13771 (PMC4563554; doi:10.1038/srep13771)
Supplement: Supplementary Information [file srep13771-s1.pdf]

Graphene-Nanodiamond Heterostructures and their application to High Current Devices

Fang Zhao, Andrei Vrajitoarea*, Qi Jiang, Xiaoyu Han, Aysha Chaudhary, Joseph O. Welch, Richard B. Jackman*

London Centre for Nanotechnology, Department of Electronic and Electrical Engineering, University College London, 17-19 Gordon Street, London WC1H 0AH, United Kingdom.

Correspondence and requests for materials should be addressed to RBJ. (Email: r.jackman@ucl.ac.uk)

*These authors contributed equally to this work.

1. Raman:

Supplementary Table 1 shows the positions for different peaks to support the research the upshift and intensity ratio.

Supplementary Table 1. Intensities for different peaks

Sample		D peak	G peak	2D peak
**Gr-ND**	**Peak position**		1586.2	2698.2
	**Intensity**		2308.9	2466
**Gr-HND**	**Peak position**	1347.1	1585.7	2690.2
	**Intensity**	3958.3	6328.6	7430.9

Supplementary Figure 1 shows the Raman results for graphene and SCD hybrid structures. It is hard to analysis D peak for GrHSCD hybrid structures compared with graphene-ND heterostructures. Due to strong D peak from crystal diamond, C-H stretching effect is very weak. ND is very complex structure compared with single crystal diamond, which is a sp^3 diamond core coated by graphitic shell or amorphous carbon with dangling bands. From Raman spectrum of SCD, no clear 2D peak present for GrSCD sample. The G peaks of GrSCD and GrHSCD have upshifts to 1583 cm^{-1} due to the doping of graphene. The ratio of I_{2D}/I_G for GrHSCD is about 0.2, which suggests high doping.

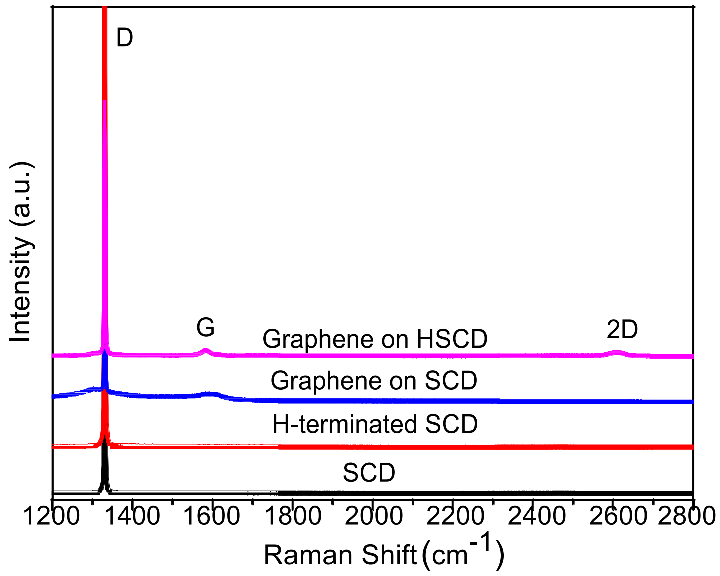

Supplementary Figure 1. Raman spectra of untreated SCD, H-SCD, graphene-SCD and graphene-HSCD.

2. XPS:

Supplementary Table 2 gives the intensity area for sp^2 and sp^3 peaks of two heterostructure and calculate the hydrogen coverage.

Supplementary Table 2.Intensity area for sp^2 and sp^3 peaks

Sample	sp^2	sp^3	θ	θ/(1+θ)
**Gr-ND**	64137.23	14852.65	0.23	19%
**Gr-HND**	25508.2	15415.4	0.61	37%

3. Impedance spectroscope for different temperatures:

Impedance spectroscopy is a useful tool for investigating the electric and dielectric behaviour of electronic or mixed conductor ceramic materials[1]. This method involves measuring the impedance as a function of frequency by applying a sinusoidal input voltage and measuring the output current. The impedance Z as a function of angular frequency ω can be written in the following complex form:

$$Z(\omega) = Z'(\omega) + jZ''(\omega) \quad (1)$$

$$Z'(\omega) = \sum_{i=1}^{n} \frac{R_i}{1+\omega^2 R_i^2 C_i^2} \quad (2)$$

$$Z''(\omega) = \sum_{i=1}^{n} \frac{\omega R_i^2 C}{1+\omega^2 R_i^2 C_i^2} \quad (3)$$

Where Z'(ω) is the real part of the impedance, associated to the resistive contribution of the measured sample, whereas Z''(ω) is the imaginary part of the impedance associated to the capacitive contribution. The variable n used in the summation can take integer values between 1 and 3, corresponding to the different type of conduction path[2]. By plotting the real part against the imaginary part of the impedance as a function of frequency we get the so-caller Cole-Cole plot. The Cole-Cole plot is a very using tool for determining the conduction mechanism in the material by fitting an equivalent circuit[2]. The most dielectric simple model consists of a resistor in series with a RC time constant which is associated to a

semicircle in the Cole-Cole plot.

The graphene-H-terminated ND sample was analysed using an AC impedance analyser with two Au/Ti (300 nm/10 nm) contacts at vacuum level. The frequency scale is from 0.1 Hz to 10 MHz. The measurement was taken from a range of temperature from room temperature to 500 ^{o}C. Increasing the temperature up to 125 ^{o}C, the results show that the radius for the low frequency semicircle increases while the high frequency semicircle shows little change in shape (Supplementary Fig. 2a). If we assign the low frequency semicircle to the graphene surface conduction path, we would expect the conductivity to decrease owing to the evaporation of the water layer on the surface.

At this temperature we would expect negligible modifications in the graphene-ND interface, so the high frequency semicircle may be associated to this conduction path. At higher temperatures from 150 ^{o}C to 250 ^{o}C both semicircles start to decrease. For the low frequency semicircle this might be due to the elimination of residues or remaining PMMA on the graphene surface, which would explain its increasing conductivity. Whereas for the high frequency semicircle, this can be explained in terms of a possible activation of the hydrogen bonds in the graphene-ND interface. Further increasing the temperature the high frequency semicircle decreases at a higher rate than the other, and results show that after 275 ^{o}C we have only one semicircle that further decreases up to 500 ^{o}C (Supplementary Fig.2c). The low frequency semicircle after 400 ^{o}C is too small to show. The sheet resistances are given for the two semicircles as the sample's temperatures are increased in supplementary Table 3. At these temperatures we would expect breaking of C-H bonds between the graphene-ND interface, which would ultimately eliminate its associated conduction path leaving only the graphene surface conduction path.

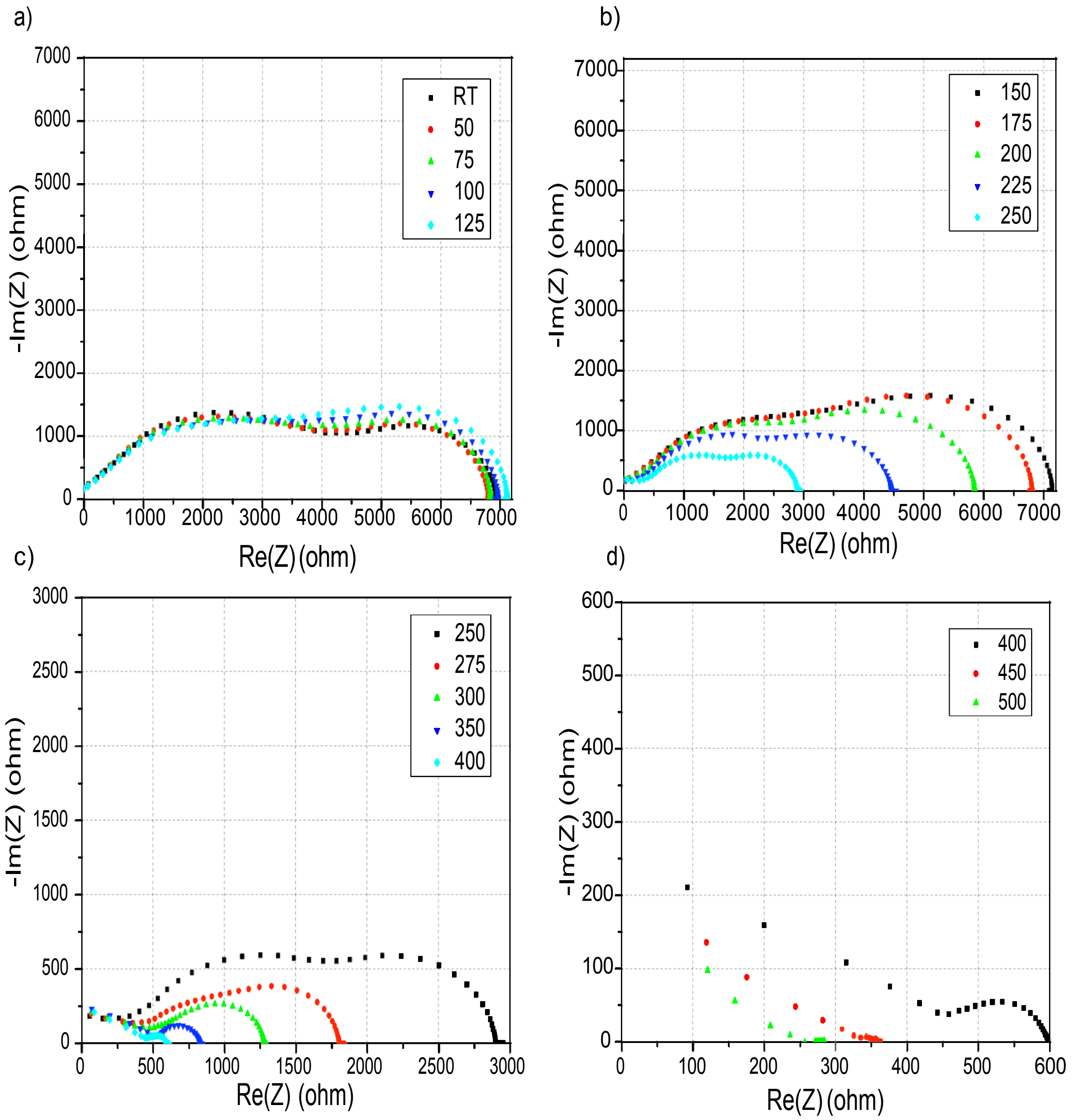

Supplementary Figure 2. Impedance spectra of graphene on H-terminated ND heterostructure for different temperature. (**a**) room temperature to 125 °C. (**b**) 150 °C to 250 °C. (**c**) 250 °C to 400 °C. (**d**) 400 °C to 500 °C

Supplementary Table 3. Sheet resistances for different temperatures

Conduction Paths	Low Frequency (Graphene)	High Frequency (C-H)
**Temperature (C)**	**R(ohm)**	**R(ohm)**
**Room T**	2898.5	4550.7
**50**	2914.9	4671.2
**75**	2972.1	4909.1
**100**	3070.6	5249.9
**125**	3375.3	5541.3
**150**	3599.3	5554.5
**175**	3468.6	4902.6
**200**	2837.4	3953.6
**225**	2272.8	3022.3
**250**	1350.1	1911.2
**275**	896.8	1702.6
**300**	604.7	--
**325**	424.6	--
**350**	296.5	--
**400**	135.1	--
**450**	8.2	--
**500**	--	--

4. Hall effect measurement for different diamond substrates samples

The measured values for the carrier density, mobility, sheet resistivity and doping type, done in room temperature conditions, are recorded in Supplementary Table 4. These measurements were done for CVD graphene transferred on both SCD and ND thin films, where the diamond substrates have been patterned with both hydrogen and oxygen surface terminations. The surface roughness of diamond surface plays an important role in reducing electron scattering. H-terminated SCD AMF images are show in Supplementary Fig.3. The first result to recognize is the fact that pristine graphene transferred on both diamond platforms with both H and O monolayer attachments lead to a p-type system. This can be also confirmed for hydrogenated graphene as well. In the case of weakly attached adsorbates, these act as donors or acceptors that ultimately change the graphene carrier concentration[3]. In the case of H and O species, these act as donors that transfer the graphene electrons from the surface to the diamond or water layer interface, giving rise to p-type graphene. The O species have higher donor ability than the H species, which can be explained in terms of a higher induced carrier

density.

In terms of carrier mobility measurements two main results can be distinguished. Firstly, the maximum mobility is associated to the H terminations on the diamond surface, corresponding to the lowest carrier mobility. As explained by Avouris[4], an important factor affecting the carrier mobility in graphene is the carrier density, where if you increase the density in monolayer graphene you decrease the mobility, a behavior depending on the dominant scatterers[4], . Therefore, a decrease in mobility for the case of O terminations can be associated to the increase in carrier density compared to the case of H terminations. The second important result to be mentioned is the fact that mobility values for both graphene on hydrogenated SCD and ND films are close, of the same order of magnitude. It is to be expected that graphene on SCD to have better, if not the best, transport properties considering the polished SCD surface has a very low surface roughness compared to the roughly prepared ND film. Yet the SCD platform is a very expensive approach for commercial applications, and a low cost diamond substrate is desirable. Effectively these results show that our prepared ND films are a less expensive platform for graphene, with the prospect of low cost carbon sp^2-on-sp^3 technology[5].

Supplementary Table 4. Hall Effect measurement results for different samples

Sample	Doping	Resistance (kΩ/sq)	Carrier density (cm^{-2})	Mobility (cm^2/Vs)
**Gr-SiO_2-Si(reference)**	P type	1.05	4.48×10^{13}	132.65
**Gr-H-SCD**	P type	4.39	6.44×10^{12}	220.86
**Gr-O-SCD**	P type	1.34	2.79×10^{13}	195.5
**Gr-H-ND**	P type	2.65	1.09×10^{13}	214.9
**Gr-O-ND**	P type	2.51	5×10^{13}	50
**Gr-ND**	P type	4.43	4×10^{13}	33.86
**Gr-H-ND (after annealing)**	P type	2.78	3.96×10^{13}	56.56

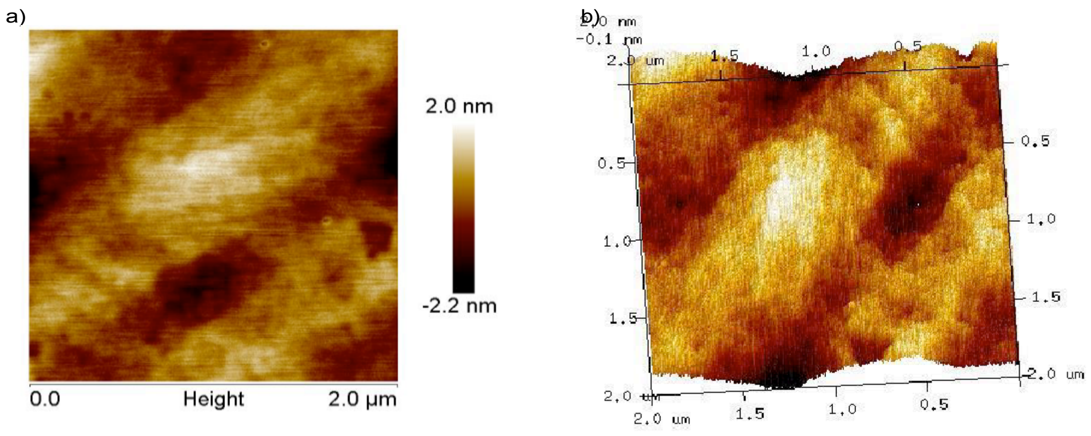

Supplementary Figure 3. AFM images of top-view (**a**) and 3D-view (**b**) of hydrogen terminated ND surface.

5. Graphene/Ti and Graphene/WC contacts analysis

Due to the excellent adhesive capability of Ti in carbon-based devices, a Ti/Au contact electrode was described as a lower contact resistivity in the units of either Ωμm or Ωμm2 [6] . From Ref [7], it has been shown the work function of the graphene-Ti contact shifted by altering the metal thickness, which suggests the work function of bulk Ti (thickness >4 nm) as 4.29 eV. In our case, 10 nm Ti was deposited on the graphene. The work function of Ti has been determined by Kelvin Probe, which is 4.3 eV. In FIB system, WC is the only available contact source, which the atomic ration is W: C: O: Ga = 31: 54: 8: 7[8], which is with a low electrical resistivity ($\sim 2 \times 10^{-7}$ Ωμm)[9]. It has been shown that WC can be stably attached on the carbon surface[10]. The work-function of WC contact, determined by Kelvin Probe, is about 4.7 eV, which is same with that in Ref [8]. Due to the difference of work function between graphene and metals, electrons are transferred from one to the other to equilibrate the Fermi levels. The work function for graphene, Ti and WC are 4.5, 4.3, and 4.7 eV, respectively[6]. The differences of work function for graphene/Ti and graphene/WC were same. The supplementary Fig.4 shows the band diagrams for graphene/Ti and graphene/WC interface. The work function of Ti was 4.3 eV, which was smaller than the work function of graphene.

After the deposition of Ti, the graphene-Ti interfaces were formed, the Fermi levels of both Ti and graphene would be coupled. As the Fermi level in Ti was higher than that in graphene, electrons transferred from Ti to graphene and the Fermi level of graphene shifted upwards compared with the Dirac point, as shown in supplementary Fig.4(a). While the work function of WC was 4.7 eV, which was higher than that of graphene. Electrons moved from graphene to WC and the Fermi level of graphene shifted downwards compared to the Dirac point, as shown in supplementary Fig.4(b). Both of Ti and WC contacts had lower resistance and same difference of work functions.

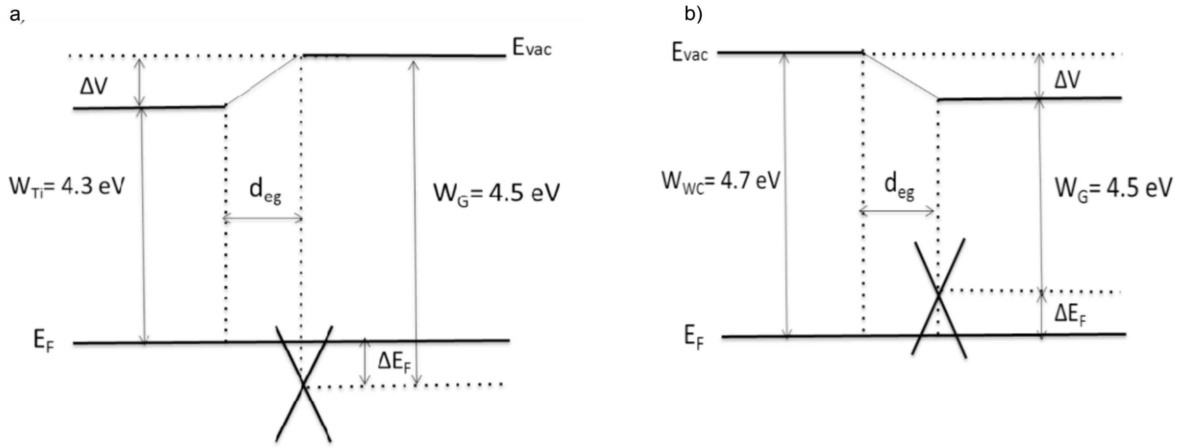

Supplementary Figure 4. The comparison of band diagrams for graphene/Ti (a) and graphene/WC (b). The vacuum energy level (E_{vac}), the difference in the work function between metal and graphene (ΔV), the equilibrium interfacial distance (d_{eq}), the Fermi level (E_F), the difference in the energy levels between the Dirac point and Fermi level of graphene (ΔE_F).

Reference:

1. Macdonald, J. Impedance spectroscopy: Models, data fitting, and analysis. *Solid State Ionics*, **176,** 1961–1969, (2005).

2. Conway, B. E. in *Impedance Spectroscopy: Theory, Experiment, and Applications*, 2nd Edn. (eds Barsoukov E. & Macdonald J.R) Ch. 4, 475-477(John Wiley & Sons, 2005)

3. Zhao, F. *et al.* Electronic properties of graphene-single crystal diamond heterostructures. *J. Appl. Phys.*, **114**, 053709, (2013).

4. Avouris, P. Graphene: Electronic and Photonic Properties and Devices. *Nano Lett.*, 4285–4294,(2010).

5. Yu, J., Liu, G., Sumant, A. V, Goyal, V. & Balandin, A. a. Graphene-on-diamond devices with increased current-carrying capacity: carbon sp^2-on-sp^3 technology. *Nano Lett.*, **12**, 1603–8 (2012).

6. Nagashio, K. et al. Contact resistivity and current flow path at metal/graphene contact. *Appl. Phys. Lett. 97, 143514 (2010).*

7. Yang, S. et al. Direct obsercation of the work function evolution of graphene-two-dimensional metal contacts. *J. Mater. Chem. C*, 2, 8042-6 (2014).

8. Kometani, R. and Ishhara, S. Nanoelectromechanical device fabrications by 3-D nanotechnology using focused-ion beams. *Sci. Tcchnol. Adv. Mater.* 10, 034501 (2009).

9. Pierson, H. O. in *Handbook of chemical vapor deposition: Principles, Technology, and Applications.* (eds Pierson, H. O.) Ch. 9, 253-254 (William Andrew Inc. 1999).

10. Chen, W. F.,et al.Tungsten Carbide-Nitride on Graphene Nanoplatelets as a Durable Hydrogen Evolution Electrocatalyst. *ChemSusChem*, 7, 2414–2418 (2014).
